# Preparation, Characterization, and Biological Evaluation of a Hydrophilic Peptide Loaded on PEG-PLGA Nanoparticles

**DOI:** 10.3390/pharmaceutics14091821

**Published:** 2022-08-29

**Authors:** Lisa Marinelli, Michele Ciulla, Jeffrey A. S. Ritsema, Cornelus F. van Nostrum, Ivana Cacciatore, Marilisa Pia Dimmito, Ferdinando Palmerio, Giustino Orlando, Iole Robuffo, Rossella Grande, Valentina Puca, Antonio Di Stefano

**Affiliations:** 1 Department of Pharmacy, University “G. d’Annunzio” of Chieti-Pescara, 66100 Chieti, Italy; 2 Department of Pharmaceutics, Utrecht Institute for Pharmaceutical Sciences (UIPS), Utrecht University, Universiteitsweg 99, P.O. Box 80082, 3508 TB Utrecht, The Netherlands; 3 Institute of Molecular Genetics, National Research Council, Section of Chieti, 66100 Chieti, Italy; 4 Center for Advanced Studies and Technology (CAST), University “G. d’Annunzio” of Chieti-Pescara, 66100 Chieti, Italy

**Keywords:** double emulsion, nanoprecipitation, PEG-PLGA, polymeric nanoparticles, quorum sensing inhibitors, RNAIII inhibiting peptide, *S. aureus* biofilm

## Abstract

The encapsulation of peptides and proteins in nanosystems has been extensively investigated for masking unfavorable biopharmaceutical properties, including short half-life and poor permeation through biological membranes. Therefore, the aim of this work was to encapsulate a small antimicrobial hydrophilic peptide (H-Ser-Pro-Trp-Thr-NH_2_, FS10) in PEG-PLGA (polyethylene glycol-poly lactic acid-co-glycolic acid) nanoparticles (Nps) and thereby overcome the common limitations of hydrophilic drugs, which because they facilitate water absorption suffer from rapid degradation. FS10 is structurally related to the well-known RNAIII inhibiting peptide (RIP) and inhibits *S. aureus* biofilm formation. Various parameters, including different method (double emulsion and nanoprecipitation), pH of the aqueous phase and polymeric composition, were investigated to load FS10 into PEG-PLGA nanoparticles. The combination of different strategies resulted in an encapsulation efficiency of around 25% for both the double emulsion and the nanoprecipitation method. It was found that the most influential parameters were the pH—which tailors the peptides charge—and the polymeric composition. FS10-PEG-PLGA nanoparticles, obtained under optimized parameters, showed size lower than 180 nm with zeta potential values ranging from −11 to −21 mV. In vitro release studies showed that the Nps had an initial burst release of 48–63%, followed by a continuous drug release up to 21 h, probably caused by the porous character of the Nps. Furthermore, transmission electron microscopy (TEM) analysis revealed particles with a spherical morphology and size of around 100 nm. Antimicrobial assay showed that the minimum inhibitory concentration (MIC) of the FS10-loaded Nps, against *S. aureus* strains, was lower (>128 µg/mL) than that of the free FS10 (>256 µg/mL). The main goal of this work was to develop polymeric drug delivery systems aiming at protecting the peptide from a fast degradation, thus improving its accumulation in the target site and increasing the drug-bacterial membrane interactions.

## 1. Introduction

For a number of therapeutic peptides under development, unfavorable physicochemical and pharmaceutical properties limit their translation to pharmaceuticals. Regarding intravenous administration, the pharmacological activity of therapeutical peptides can be adversely affected by chemical and enzymatic instability, rapid in vivo elimination, inadequate permeation through physiological membranes, and limited shelf life [1,2].

Nanoparticulate systems arouse interest because of their extensive applications in different medical fields [3,4,5]. They have been applied to enhance the delivery of drugs due to their capability to protect the payload [6,7,8]. Particularly, nanoparticles (Nps) based on poly(D,L-lactic-co-glycolic acid) (PLGA) have been extensively investigated as delivery vehicles for peptide-based drugs. They have good biocompatibility and their formulations have been approved by the Food and Drug Administration (FDA) and the European Medicines Agency (EMA) [9,10,11,12,13]. PLGA-based nanoparticles have several attractive characteristics such as improving drug stability, ability to modulate drug release, the possibility for surface modification, and tunable degradation properties depending on the polymer composition and molecular weight [14].

The hydrophobicity of PLGA results in the good encapsulation and loading efficiency of hydrophobic compounds. For these compounds, drug-loaded-PLGA nanoparticles and microparticles are prepared by dissolving the hydrophobic drug and the polymer in a volatile water-immiscible solvent such as dichloromethane (DCM). The obtained solution is then poured into an excess of water in which a surfactant is dissolved to obtain an oil-in-water emulsion after stirring the two-phase system. Drug-loaded nanoparticles are subsequently obtained after evaporation of the volatile organic solvent [15,16]. Double emulsion and nanoprecipitation methods are used to load hydrophilic drugs, including peptides and proteins, in PLGA-based nano-and microparticles [17,18].

In the double emulsion method, the hydrophilic drug is dissolved in a small amount of water, mostly containing a surfactant, which is subsequently added to a solution of PLGA, e.g., DCM. After stirring, the resulting water-in-oil emulsion is dispersed into a larger amount of water to obtain a water-in-oil-in-water emulsion [19,20].

Nanoprecipitation encompasses the dropwise addition of a solution of PLGA and drug in a water-miscible solvent (e.g., acetone, DMF) in a large excess of water under vigorous stirring. Nanoparticles are formed due to solvent exchange resulting in precipitation of PLGA by which process the drug is entrapped [21,22].

For both methods, Nps are recovered by centrifugation after the removal of the organic solvent. Encapsulation efficiencies are not always high but can be optimized by varying the formulation and process parameters [23,24,25,26].

**Figure 1:**
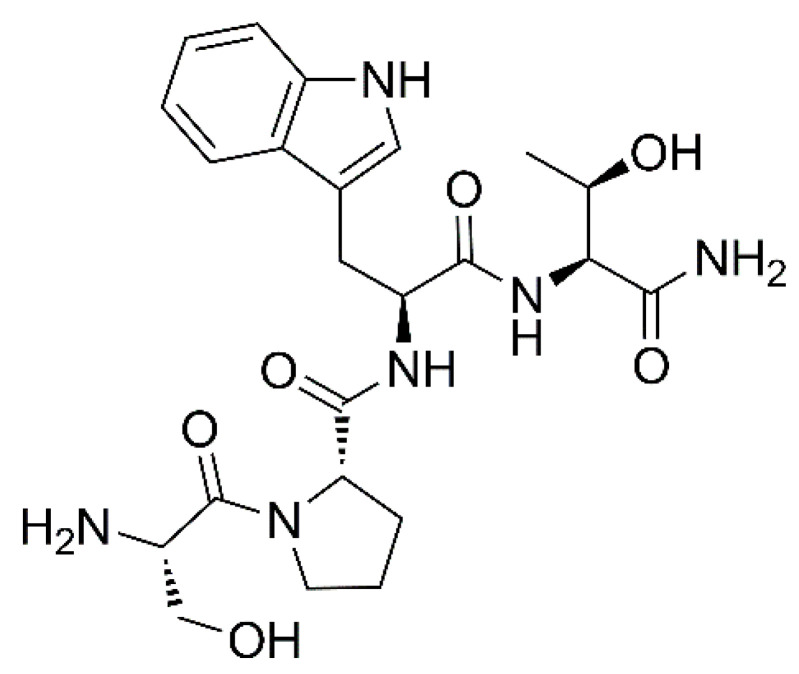
Chemical structure of FS10.

The aim of this work was to explore the encapsulation of FS10, a small synthetic hydrophilic peptide, in PEG-PLGA Nps with the main objective to (i) protect the selected peptide possessing simple amide bonds, susceptible to peptidases; (ii) improve its accumulation in the target site; and (iii) promote the drug-bacterial membrane interaction, a process normally restricted for hydrophilic drugs against gram-positive bacteria lacking porins which act as hydrophilic transmembrane channels. FS10 is a tetrapeptide composed of a linear sequence of H-Ser-Pro-Trp-Thr-NH_2_ (Figure 1), structurally related to the RNAIII inhibiting peptide (RIP) and able to inhibit *S. aureus* biofilm pathogenesis interfering in the Quorum Sensing mechanism (QS) [27,28,29]. This QS process is of essential importance for the communication of bacterial pathogens, allowing them to exchange and share information. It has been shown that QS inhibition results in a reduction in the adhesion capacity of bacteria and in blockage of the synthesis of bacterial toxins [30]. Notably, there are different approaches potentially useful to hinder biofilm formation including biological and physico-chemical mechanisms [31,32,33,34].

To develop a formulation suitable to efficiently load and release FS10 in PEG-PLGA Nps, both the double emulsion and the nanoprecipitation method were considered.

The effect of the different methods and formulation parameters on Nps physico-chemical properties (Z-average diameter, polydispersity, ζ-potential), entrapment efficiencies, loading capacities, in vitro release kinetics, and morphological features, were investigated. Furthermore, the antimicrobial and antibiofilm activity of FS10-PEG-PLGA Nps against *S. aureus* strains were studied.

## 2. Materials and Methods

### 2.1. Materials

FS10 as TFA salt was purchased from GenScript (Piscataway, NJ, USA). N-cyclohexyl-3-aminopropanesulfonic acid (CAPS), (4-(2-hydroxyethyl)-1-piperazineethanesulfonic acid) (HEPES), phosphate buffer saline (PBS, pH 7.4), poly(vinyl alcohol) (PVA; MW 30,000–70,000; 88% hydrolyzed), tin(II) 2-ethylhexanoate (Sn(Oct)2) and poly(ethylene glycol) monomethyl ether (MePEG5000) were obtained from Sigma Aldrich (St. Louis, MO, USA). D,L-lactide, glycolide, and poly(lactic-co-glycolic acid) (25 kDa uncapped PLGA, lactide:glycolide 50:50) were obtained from Corbion (Amsterdam, The Netherlands). N,N-dimethylformamide (DMF), dichloromethane (DCM), chloroform, diethyl ether, and acetonitrile (ACN) were purchased from Biosolve (Valkenswaard, The Netherlands). Toluene obtained from Acros (Beerse, Belgium), was stored over 3 Å molecular sieves (Sigma Aldrich, St. Louis, MO, USA). Unless otherwise stated, all chemicals were used as received.

### 2.2. Synthesis of PEG-PLGA

Random copolymer of glycolide and D,L-lactide was synthesized by ring-opening polymerization in the melt using MePEG_5000_ as initiator and Sn(Oct)_2_ as catalyst [35]. Briefly, D,L-lactide (4.45 g), glycolide (3.58 g) and dried MePEG_5000_ (3.09 g) were loaded into a Schlenk tube followed by the addition of Sn(Oct)_2_ (12.5 mg) in dry toluene. After the removal of toluene by applying vacuum, the Schlenk tube was closed and subsequently transferred into an oil bath at 130 °C. The melt polymerization proceeded overnight and, after cooling to room temperature, the crude product was dissolved in chloroform, precipitated into an excess of diethyl ether, and vacuum dried overnight.

### 2.3. Polymer Characterization

The number average molecular weight, PEG, and the molar ratio of lactide to glycolide of the obtained PLGA-PEG block copolymer were determined by ^1^H-NMR (Gemini-300 MHz spectrometer at 298 K). The molar % of composing units (lactic acid (%L), glycolic acid (%G) was determined according to the following formulas [36]:

I_G_ = I_4.8–4.9_/2

I_L_ = (I_1.5_/3 + I_5.1–5.2_)/2

% L = (I_L_/I_L_ + I_G_) × 100

% G = (I_G_/I_L_ + I_G_) × 100

where I_G_ and I_L_ are the peak integrals per one proton of each monomer unit, and I_n_ are the integrals obtained from the NMR spectra at the indicated peak shifts (ppm). The number average molecular weight (M_W_) of the diblock copolymer is given by: (I_L_/I_PEG_ × M_W_ of lactic acid unit) + (I_G_/I_PEG_ × M_W_ glycolic acid unit) + M_W_ MePEG_5000_; I_PEG_ is the peak integrals per one proton of PEG. The weight %PEG of the block copolymer is calculated by dividing the molecular weight of MePEG_5000_ with the calculated diblock molecular weight × 100.

The relative molecular weight and molecular weight distribution of the obtained polymer were determined using GPC (Waters Alliance system). Two PL-gel 5 μm Mixed-D columns fitted with a guard column (Polymer Labs, MW range 0.2–400 kDa) were used and calibration was done using polyethylene glycol standards with narrow molecular weight distributions. A solution of 10 mM LiCl in DMF was used as the mobile phase and the elution rate was 1 mL/min. Detection was done using a Waters 2414 Refractive Index detector.

The thermal properties of the block copolymer were analyzed using differential scanning calorimetry (DSC Q2000, TA Instruments, New Castle, DE, USA). Approximately 5 mg polymer sample was loaded into an aluminum pan, and after heating from room temperature to 120 °C, with a heating rate of 10 °C/min, the sample was cooled down to −50 °C. Thereafter, the sample was heated to 120 °C with temperature modulation at ± 1 °C and a ramping rate of 2 °C/min. The second cycle was used to determine the glass transition temperature (T_g_) of the synthesized polymer.

### 2.4. Nanoparticles Preparation

**Figure 2:**
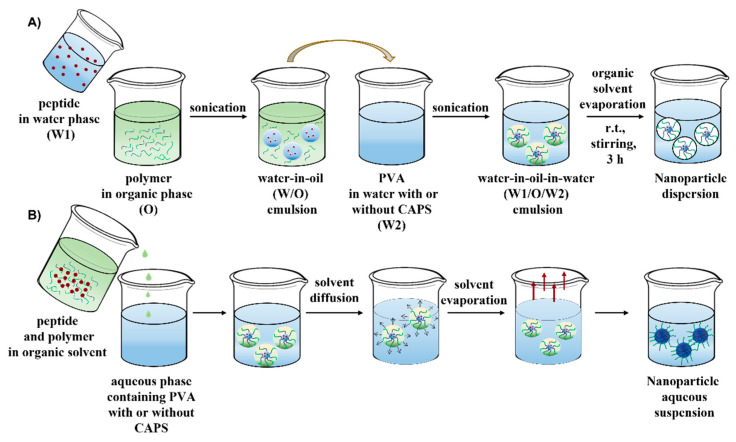
FS10-loaded Nps prepared by (**A**) double emulsion and (**B**) nanoprecipitation methods.

Both a double emulsion solvent evaporation technique and nanoprecipitation method were chosen to prepare PLGA nanoparticles loaded with the FS10 peptide (Figure 2). Nanoparticles obtained by a double emulsion solvent evaporation technique were prepared according to Zambaux et al., with slight modifications [37]. Briefly, a solution of peptide in reverse osmosis water (300 μL, 5 mg/mL) was emulsified in 3 mL of DCM containing different blends of mPEG_5000_-PLGA and PLGA (lactide:glycolide 50:50, total polymer concentration was 5% *w*/*v*) in an ice-bath using a sonifier (S-450A, Branson Ultrasonics Corp., Danbury, CT, USA) for 1 min at 10% of amplitude. The *w/o* emulsion was subsequently emulsified into an external aqueous PVA solution (30 mL, PVA 0.5% *w*/*v*) with or without buffer (N-cyclohexyl-3-aminopropanesulfonic acid, CAPS, 5 mM, pH 9.7), in an ice-bath using the same sonicator for 2 min at 10% of amplitude to form a water-in-oil-in-water (*w*/*o*/*w*) emulsion. Next, DCM was evaporated at room temperature under vigorous stirring for 3 h.

Nanoparticles obtained by nanoprecipitation were prepared according to Bilati et al. [38]. In short, blends of mPEG_5000_-PLGA/PLGA and peptide were weighed and dissolved in ACN (1 mL). The organic phase was added dropwise into 20 mL of a PVA 0.5% solution with or without pH control (CAPS 5 mM, pH 9.7) and stirred magnetically for 3 h at room temperature until ACN evaporation.

The Nps obtained both with the double emulsion and nanoprecipitation methods were collected by ultracentrifugation (40,000× *g* for 30 min) and washed twice with 20 mL reverse osmosis water. Nps obtained from both procedures were lyophilized and stored at −20 °C. All formulation batches were prepared in triplicate.

### 2.5. Characterization of Empty and FS10-Loaded Nanoparticles

Loaded and unloaded Nps were dispersed in distilled deionized water (final concentration 50 µg/mL) and their Z-average size and size distribution were measured using Dynamic Light Scattering (DLS; Zetasizer 4000, Malvern Instruments, Malvern, UK) at 25 °C at an angle of 90°. The ζ-potential of the nanoparticles, suspended in 5 mM HEPES solution (pH 7.4, final concentration 50 µg/mL), was determined by laser Doppler electrophoresis using a Zetasizer Nano-Z (Malvern Instruments Ltd., Malvern, UK).

### 2.6. Protein Entrapment Efficiency and Loading %

Protein entrapment efficiency and loading percentage were determined according to Yang et al. and Ravivarapu et al. with slight modifications [39,40]. In brief, freeze-dried nanoparticles were accurately weighed (30 mg), dissolved in 1 mL of DCM, sonicated using an ultrasonic homogenizer for 30 s at 10% of amplitude, and stirred for 1 h at room temperature. Subsequently, 3 mL water was added to reach a concentration of 10 mg/mL of polymer, and the dispersion was stirred at 40 °C for 2 h to evaporate DCM. Next, the precipitate PLGA was removed by centrifugation (30 min at 20,000× *g*) and the aqueous solution was filtered through a 0.2 µm filter. The amount of protein in the supernatant was measured by HPLC. This method was validated by the addition of a known amount of peptide to a DCM solution of empty nanoparticles, followed by the addition of water, centrifugation, and filtration as described above (98.9% of recovery). Protein entrapment efficiency is defined as the amount of protein entrapped divided by the feed protein × 100%. The protein loading % is calculated as the encapsulated amount of protein divided by the dry weight of the loaded particles × 100%. HPLC system was a Waters 600 HPLC pump (Waters Corporation, Milford, MA, USA), provided with a Waters 2996 photodiode array detector, a 20 μL Rheodyne injector loop, and a computer-integrating apparatus. Chromatographic separation was achieved using a BEH C18 Column, 3.5 µm, 2.1 mm × 100 mm, using a linear elution gradient starting at 100% solvent A (95% H_2_O, 5% ACN, and 0.1% trifluoroacetic acid) to 0% solvent A and 100% solvent B (100% ACN and 0.1% trifluoroacetic) over 4 min, followed by re-equilibration to 100% solvent A in 4 min. The flow rate was 0.25 mL/min, and the detection was performed at 280 nm. FS10 standard solutions (5–100 μg/mL) were used for the calibration. The stock standard solution of FS10 was prepared in the mobile phase at the concentration of 500 µg/mL. The calculated LOD and LOQ for our analytical method, previously validated according to the ICH guidelines, were 3 and 4.5 μg/mL, respectively.

### 2.7. In Vitro Release of FS10 from PLGA Nps

Freeze-dried nanoparticles were suspended in sodium phosphate buffer (PBS, 0.033 M NaH_2_PO_4_, 0.066 M Na_2_HPO_4_, 0.056 M NaCl, pH 7.4) and samples of 1 mL of the homogeneous suspension (final concentration 10 mg/mL) were aliquoted into 1.5 mL Eppendorf tubes. The aliquots were incubated at 37 °C under mild agitation. At different time points, one tube was taken, the particles were centrifuged at 20,000× *g* for 30 min and the amount of peptide in the supernatant was measured by HPLC (see Section 2.6).

To better evaluate the drug release mechanism and kinetics, the drug release data, obtained from various in vitro release experiments, were fitted to different kinetics models, including the zero-order, first-order, and Higuchi.

### 2.8. Morphology Evaluation by Transmission Electron Microscopy

To visualize MLNs, negative staining was performed. Formvar and carbon-coated copper grids (200 mesh; TAABLab.UK) were floated, film side down, on 20 μL of each sample for 20 s. Then the grids were stained with uranyl acetate [20 μL of 2% (*w*/*v*)] and gently touched to filter paper to remove excess sample/water. The specimens were imaged using a transmission electron microscope Philips 268 D (FEI, Eindhoven, The Netherlands).

### 2.9. Determination of Minimum Inhibitory Concentration (MIC) and Minimum Bactericidal Concentration (MBC) of FS10, FS10-Loaded-NpS, and Empty NpS versus S. aureus ATCC 43300

*Staphylococcus aureus* ATCC 43300 methicillin and oxacillin resistant, has been used in the study. *S. aureus* ATCC 43300 was cultivated as described before [41,42]. In brief, *S. aureus* was stored at −80 °C before being thawed at room temperature and plated on Tryptone Soya Agar (TSA; Oxoid Ltd., Hampshire, UK) for 24 h at 37 °C. Subsequently, the microorganism was grown in Mueller Hinton Broth (MHB; Oxoid Ltd.) for 16 h at 37 °C under shaking conditions at 125 rpm. The overnight broth culture was diluted in MHB 2 (MHB2, Cation-adjusted, Millipore, Merck, KGaA, Darmstadt, Germany) to reach an optical density of 550 nm (OD550, Spark^®^ multimode microplate reader, Tecan Trading AG, Switzerland) of 0.4 corresponding to 2–8 × 10^8^ Colony Forming Unit (CFU)/mL. The broth culture was then diluted 1:100 in MHB 2 and used for the evaluation of the MIC in the 96-well plate. Each well contained the bacteria at a final concentration of 2–8 × 10^5^ CFU/mL.

The MIC and MBC were determined in MHB 2 by using the broth microdilution method according to the guidelines of the Clinical & Laboratory Standards Institute [43].

The three test compounds, FS10, FS10-loaded-Nps, and empty Nps were diluted in ultrapure water and then further diluted in MHB 2 to reach the range concentrations of 0.5–512 µg/mL. Controls, consisting of (i) *S. aureus* broth culture in MHB 2 without the addition of FS10, FS10-loaded-Nps, and empty Nps; (ii) MHB 2 with FS10; (iii) MHB 2 with FS10-Loaded-Nps; (iv) MHB 2 with Empty Nps and (v) just MHB 2 were added. Two independent experiments were performed in triplicate. The plates were incubated at 37 °C for 24 h. The MIC was defined as the lowest concentration of FS10, FS10-Loaded-Nps, and empty Nps without visible growth.

### 2.10. FT-IR

An infrared spectroscopy study was performed using a Spectrum TWO FT-IR (Perkin Elmer, Waltham, MA, USA). Spectra were recorded in the range of 4000–400 cm^−1^, with a resolution of 4. Samples were compressed by applying pressure to obtain pellets of the free FS10, empty PEG-PLGA Nps, and FS10-loaded PEG-PLGA Nps.

### 2.11. In Vitro Stability Studies

Blank and FS10-loaded PEG-PLGA Nps were lyophilized, and the resulting product was studied for long-term stability (14 days) at 4 °C. Nanoparticulate stability was evaluated for changes in size, PDI, and entrapment efficiency. After certain days of storage, three samples of each lyophilized NPs preparation were reconstituted and were characterized by DLS.

### 2.12. Determination of the Minimum Biofilm Inhibitory Concentration (MBIC) of FS10, FS10-Loaded-Nps, and Empty Nps versus S. aureus ATCC 43300

The determination of the Minimum Biofilm Inhibitory Concentration (MBIC) was performed to evaluate a possible effect of the developed formulations as quorum sensing inhibitors. *S. aureus* biofilm was formed in TSB plus 1% glucose with the adjunct of FS10, FS10-Loaded-Nps and empty Nps at the concentrations of 64–128–256 µg/mL. *S. aureus* grown in TSB plus 1% glucose was used as a control of forming biofilm. In detail, *S. aureus* was grown in Tryptone Soya Broth (TSB; Oxoid Ltd.) for 16 h at 37 °C under shaking conditions at 125 rpm. The overnight broth culture was diluted in TSB plus 1% glucose until the OD550 of 0.4 corresponding to 2–8 × 10^8^ CFU/mL. The broth culture was then diluted until the concentration of 2–8 × 10^5^ CFU/mL in the well and used for the biofilm formation. After 24 h of incubation, the antibiofilm activity was determined by using three assays: the alamarBlue assay, the CFU count method, and the Crystal Violet assay.

In detail, at the end of the incubation, the non-adherent cells were removed, and the biofilms were washed with 100 µL of Phosphate Buffered Saline (PBS; Sigma Aldrich, St. Louis, MO, USA). AlamarBlue was diluted to 10% in the appropriate broth and 100 µL were added to each biofilm in the wells. The biofilms were incubated for 1 h and 30 min at 37 °C. At the end of the incubation, the absorbance of alamarBlue was determined by using the Spark^®^ multimode microplate reader (Tecan), then the percentage of alamarBlue reduction was determined as previously described [44].

The CFU count was carried out starting from the wells stained with alamarBlue. The biofilms were scraped, and serial dilutions of the biofilms were performed in PBS, plated on MHA, and incubated at 37 °C for 24 h.

The antibiofilm activity was also determined through the Crystal Violet staining. Biofilms were rinsed in PBS, dried for 1 h at 60 °C, and stained with 0.1% Crystal Violet for five minutes at room temperature; the Crystal Violet was removed, and the biofilms were washed with 200 µL of sterile water; then the biofilms were dried for 30 min at room temperature and decolored by pipetting with 100 µL of a water solution of 33% acetic acid (Sigma-Aldrich), for 10 min. Then the absorbance at 590 nm was measured (Spark^®^ multimode microplate reader, Tecan).

### 2.13. Hemolytic Activity

Murine erythrocytes were diluted to 6.5–7 × 10^7^ cells/mL, and aliquots (25 μL) were added to a PBS solution (2 mL) (pH 7) in the presence or without the tested sample. As a reference, lytic peptide melittin (1 mL) was added to a blood suspension (1 mL) and incubated at 37 °C for 1 h. The treated samples were centrifuged (1500× *g* for 10 min), and the collected supernatants were analyzed. The total hemoglobin released was used to measure the hemolytic activity as a function of the sample concentration. Total hemoglobin was determined by suspending the same aliquot of cells in 2 mL of distilled water and measuring the absorbance at 414 nm. All the experiments were performed in triplicate.

### 2.14. Statistical Analysis

The differences in the means of the results between untreated and treated samples were evaluated by one way ANOVA (GraphPad Software, San Diego, CA, USA) and Dunnett’s multiple comparison test. S. aureus grown in only medium was used as the control in the statistical analysis. The probability value of *p* ≤ 0.05 was considered significantly different.

## 3. Results and Discussion

### 3.1. Synthesis and Characterization of PEG_5000_-PLGA

A PEG_5000_-PLGA block copolymer was synthesized by the ring-opening polymerization of lactide and glycolide in the melt using MePEG_5000_ and stannous octoate as initiator and catalyst, respectively. The copolymer compositions as well as the number average molecular weights of the obtained diblock copolymer were determined by ^1^H-NMR (Figure S1). The molecular weights and molecular weight distribution were analyzed using GPC (Figure S2). The characteristics of the synthesized polymer showed that the composition is close to the monomer feed ratios (Table 1). Further, the number average molecular weight based on NMR (17.5 kDa) was in good agreement with the theoretical molecular weight (18 kDa). The Mn values determined using GPC are underestimated compared with the results obtained by NMR (9.6 and 17.5 kDa, respectively) which can be explained because polyethylene glycol standards were used for the calibration [45,46,47].

DSC analysis showed that the synthesized PEG_5000_-PLGA possessed a *T_g_* at 1 °C (Figure S4). A single glass transition temperature demonstrates that both polymer blocks are miscible in their solid state [13,36,48]. The full miscibility of the PEG and PLGA blocks is further supported by comparing the experimental *T_g_* value with the theoretical one calculated by the Fox equation:

$$\frac{1}{T_{g}}=\frac{W_{1}}{T_{g1}}+\frac{W_{2}}{T_{g2}}$$

In which *W*_1_/*W*_2_ and *Tg*_1_/*Tg*_2_ are the weight fractions, and the glass transition temperatures of the polymer components PEG and PLGA, respectively.

### 3.2. Preparation and Characterization of FS10-Loaded Nanoparticles

Both a double-emulsion solvent and a nanoprecipitation method were used to prepare PEG-PLGA Nps loaded with the FS10 peptide (Figure 2). The effect of the pH of the water phase and the polymer blend composition, on the characteristics of the Nps, were investigated to identify conditions that result in high encapsulation efficiency of the hydrophilic peptide.

The formulations were based on the synthesized PEG_5000_-PLGA (Table 1), PLGA, and blends of PEG_5000_-PLGA/PLGA in weight ratios of 30/70, 50/50, and 70/30.

The polymer/peptide ratio was 100/1 *w*/*w*. The terminal carboxyl group of PLGA chains may promote ionic interactions with the FS10 peptide, since at pH 7.4 it exists as 50% in the protonated form (from computational studies, see the green curve in Figure S3) and increase its incorporation into the nanoparticles, while on the other hand the PEG, due to its hydrophilic nature, in addition, to ensure stealth feature to the nanoparticles, could more effectively retain the water-soluble drug limiting its back diffusion.

**Figure 3:**
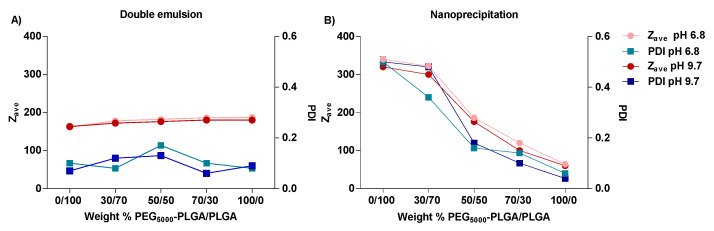
Z-average hydrodynamic diameter (Z_ave_) and polydispersity index (PDI) of FS10-loaded nanoparticles prepared by double emulsion (**A**), and nanoprecipitation method (**B**) at pH 6.8 and 9.7. All measurements were performed in triplicate (n = 3) for every single batch.

**Figure 4:**
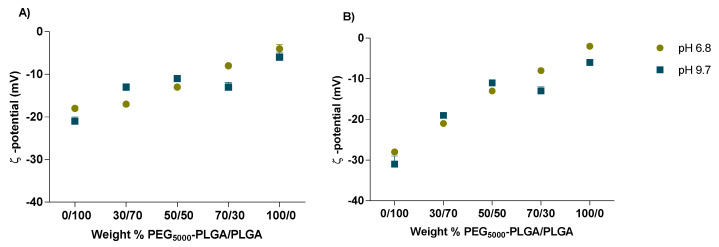
ζ-potential of FS10-loaded nanoparticles prepared by double emulsion (**A**), and nanoprecipitation method (**B**) at pH 6.8 and 9.7. All measurements were performed in triplicate (n = 3) for every single batch.

Nanoparticles prepared by the two methods were recovered with good yields ranging from 58 to 73%. The use of the PVA as surfactant resulted in the formation of small and rather uniform nanoparticles, meanwhile in the absence of PVA big aggregates and polymeric clusters were formed. No significant differences in size, PDI, and ζ-potential were observed between nanoparticles prepared at pH 6.8 and pH 9.7 (Figure 3 and Figure 4). Formulations prepared by the double emulsion method had a particle-size range from 160 to 190 nm and PDI values lower than 0.2, indicating a narrow particle size distribution (Figure 3A). A small increase in particle was observed with increasing PEG_5000_–PLGA weight fraction (from 161 to 187 nm and from 163 to 180 nm for Nps prepared at pH 6.8 and pH 9.7, respectively). Regarding the formulations prepared via the nanoprecipitation method, particle size and PDI values followed a linear decrease with increasing the PEG_5000_–PLGA weight fraction. Polymeric blends composed of 50/50 and 70/30 of PEG_5000_–PLGA/PLGA yielded nanoparticles with an average size of 176 and 100 nm, and polydispersity of 0.18 and 0.10, respectively. Indeed, the presence of a higher percentage of PEG5_000_–PLGA could suggest that the hydrophilic lattices provided by PEG stabilize the nanoparticles, thus reducing the PLGA aggregation during Nps formation [49]. An increasing PLGA fraction resulted in larger particles and high PDI values (300–322 nm and 0.48–0.36, respectively) (Figure 3B). This increased Nps size can be ascribed to the super-saturation of the system, which is important for the nucleation process [50].

With increasing PEG_5000_–PLGA weight fraction, the ζ-potential dropped from −21 to −4 mV and from −31 to −2 mV for nanoparticles prepared using the double emulsion and the nanoprecipitation, respectively (Figure 4). This can be explained by the shielding effect of PEG which masks the surface charges of the carboxylate anions of uncapped PLGA residues [51].

**Figure 5:**
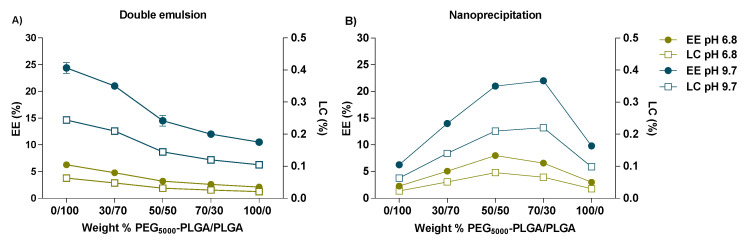
Encapsulation efficiency (EE%) and loading capacity (LC%) of FS10-loaded nanoparticles prepared by double emulsion (**A**), and nanoprecipitation method (**B**) at pH 6.8 and 9.7. All measurements were performed in triplicate (n = 3) for every single batch.

Figure 5 shows that the encapsulation efficiency of the peptide in nanoparticles, prepared by double emulsion was 6.3 and 24.4% when the external pH was 6.8 and 9.7, respectively. Computational calculations (Figure S3) showed that at pH 9.7 the peptide has no net charge and thus its solubility in the external water phase is lower than at pH 6.8 (overall charge of the peptide is positive) retarding during preparation its diffusion from the core into the external water phase [52]. For the double emulsion method, the highest peptide encapsulation was observed for formulations based on PLGA only and prepared at pH 9.7 (EE of 24.4%, Figure 5A). The reason for this high encapsulation efficiency can be explained by the ionic interaction between the ionized carboxylic end groups of the polymer and positively charged amino acid groups of the peptide [53]. Both factors favor the residence of the peptide in the inner Nps water phase where it has a higher solubility and affinity with the carboxylic end group of PLGA [54].

For Nps prepared using the nanoprecipitation method, the EE was also strongly affected by the pH of the aqueous phase. Figure 5B shows that EE increased when a certain percentage of PLGA was added to the polymeric blend, in fact for a weight % PEG_5000_-PLGA/PLGA 70/30, the maximum EE was obtained, corresponding to 22% and 8% at pH 9.7 and 6.8, respectively. However, for the formulations with an increased % of PLGA, the EE% considerably decreased, which could be explained because of the instability of the system due to a higher percentage of a hydrophobic polymer, which implies big nanoparticles and aggregates that could alter the process of encapsulation (Figure 3B).

### 3.3. In Vitro Release of FS10 from PLGA Nps

**Figure 6:**
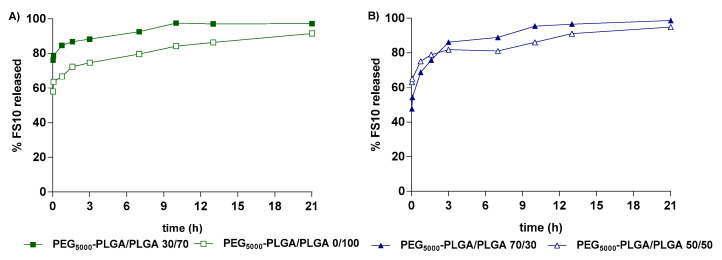
Release of FS10 peptide from different PLGA nanoparticles prepared by double emulsion (**A**) and nanoprecipitation (**B**) methods.

Nanoparticles prepared by double emulsion with blend compositions PEG_5000_-PLGA/PLGA 0/100 and 30/70, and the ones obtained by nanoprecipitation with blend ratios 50/50 and 70/30, were selected to study the in vitro release properties, because of their best physico-chemical properties in terms of size, PDI and EE%. Drug-loaded Nps showed biphasic release of the loaded FS10 peptide, suggesting that the preparation methods did not significantly affect the FS10 release (Figure 6). The different Nps showed an initial burst release of 48–63%, slightly more pronounced for nanoparticles based on PEG_5000_-PLGA/PLGA 30/70, prepared by double emulsion. Since the peptide does not or hardly dissolve in hydrophobic polymer matrices, the burst release can be explained by the diffusion of FS10 through water-filled pores which might be formed during freeze-drying of the Nps [55]. The remaining entrapped peptide is released from the Nps in the next 20 h (Figure 6).

Kinetic data showed that the in vitro release from FS10-loaded PEG-PLGA nanoparticles is best explained by the Higuchi model, except for nanoparticles based on PEG_5000_-PLGA/PLGA 50/50. R^2^ values were 0.9821 for the formulations prepared by nanoprecipitation with a blend ratio of PEG_5000_-PLGA/PLGA 70/30, while 0.9704 and 0.9885 were r^2^ values for Nps prepared by double emulsion based on PEG_5000_-PLGA/PLGA 30/70 and PEG_5000_-PLGA/PLGA 0/100, respectively. A first-order model best fitted the release of FS10 from Nps based on PEG_5000_-PLGA/PLGA 50/50 with an r^2^ value of 0.9355.

### 3.4. Morphology Evaluation by Transmission Electron Microscopy

The optimized empty and drug-loaded Nps, prepared by nanoprecipitation, were investigated by TEM analysis. Micrographs revealed nanoparticles with spherical shape and nanometric size (100 nm) in agreement with DLS characterization. Even if the obtained results are coherent with each other, smaller sizes were found for TEM-based measurements. These results can be clarified considering the technique differences since DLS method takes into account the hydrodynamic diameter of particles in suspension, while TEM images consider the gyration radius of dried particles.

**Figure 7:**
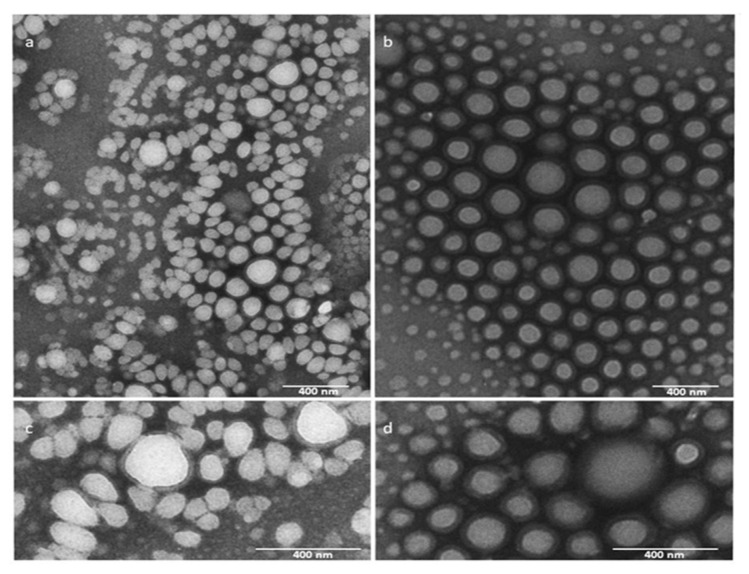
TEM images of empty Nps (blank) and FS10-loaded Nps. Empty Nps (**a**,**c**); FS10-loaded Nps (**b**,**d**). (**c**,**d**) are high magnification.

As shown in Figure 7 the empty Nps appear white (Figure 7a,c), while drug-loaded Nps appear electrondense (Figure 7b,d).

Different formulation strategies have been reported in the literature to overcome the critical issues related to the challenging delivery of hydrophilic compounds [23]. Budhian et al. improved the EE% of haloperidol up to 30% using acid terminated instead of ester capped PLGA [56]. Moreover, Imatinib mesylate was formulated in PLGA microspheres with high EE and LC by optimizing the pH of the internal (W1) and external (W2) water phases [57]. In our case the combination of different strategies allowed to increase the encapsulation efficiency of FS10, highlighting that the most influential parameters are both the polymeric blend composition, conferring the proper hydrophilic/lipophilic balance as well as the pH control, which decreases the peptide charge in the aqueous phase. As a great deal, FS10-loaded PLGA Nps have addressed the stability issue related to the drug that otherwise undergoes rapid hydrolysis (Figure S5). Moreover, even if the optimized formulations showed an immediate burst release within the first hours, they also provide a continuous drug release for 21 h. The initial high release rate may be useful to reach the therapeutical concentration of the drug suitable for managing the critical acute phase of the infection. Moreover, the following continuous release for various hours ensures the maintenance, at the infection site, of a constant concentration of FS10 useful in the late stages of the infection [58]. These release patterns may be considered beneficial for the intended antimicrobial purpose.

### 3.5. FT-IR Spectroscopy

**Figure 8:**
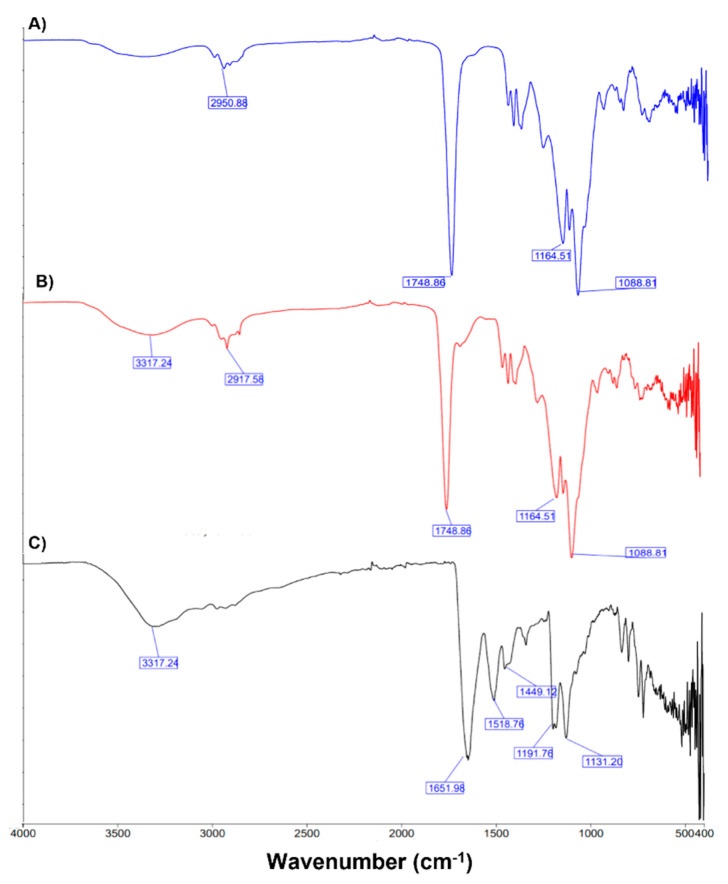
FT-IR spectra of empty PEG-PLGA Nps (**A**) FS10-loaded PEG-PLGA Nps (**B**), and free FS10 (**C**).

The FT-IR spectra were used to confirm the chemical structure of PEG-PLGA Nps. FT-IR spectra of empty PEG-PLGA Nps, FS10- loaded PEG-PLGA Nps, and the free FS10 are reported in Figure 8. Blank and loaded PEG-PLGA Nps showed as the main peak the characteristic C=O stretching of the PLGA at 1749 cm^−1^ and the C-C-O and O-C-C stretching at 1164 and 1088 cm^−1^, respectively. The spectrum of FS10 showed the NH_2_ and OH stretching at 3317 cm^−1^ and the typical amide C=O stretching at 1652 cm^−1^, whereas C=C aromatic stretching and N-C stretching are visible at 1518 and 1449 cm^−1^, respectively. Finally, C-O stretching i visible at 1191 and 1131 cm^−1^. All the typical peaks belonging to FS10 are not visible in the loaded NPs spectra, confirming the loading of the drug into the nanoparticles.

The typical peak at 3270 cm^−1^ belonging to O-H groups of PVA, which should be very strong, was not seen in the spectrum of PEG-PLGA Nps, suggesting the complete removal of PVA from the nanosystems during the washing procedure. These results confirmed that PEG was conjugated with the PLGA, and FS10 was incorporated into the Nps.

### 3.6. In Vitro Stability Studies

No significant changes in the encapsulation efficiency were observed during the 14-day storage, whereas the particle size of the formulation resulted slightly increased for both blank and FS10-loaded PEG-PLGA Nps. The PDI became much more pronounced reaching two weeks of storage, probably due to an increased difficulty in the redispersion process, which may lead to the presence of aggregates (Table 2).

### 3.7. Antibacterial Activity of FS10, FS10-Loaded-Nps, and Empty Nps versus S. aureus ATCC 43300

Notably, particle size has been shown to affect nanoparticle-bacteria interactions: the smaller the Nps size, the greater the antibacterial activity. This physico-chemical property affecting the biological activity may be related to (i) a higher propensity of lower-sized Nps to permeate into the bacterial membrane and (ii) a bigger surface area-to-mass ratio of the smaller Nps which increases both the adaption and the binding to the microbial surface. Starting from this evidence, since no significant differences between the optimized formulations were observed in terms of EE% and release profile, the FS10-loaded PLGA Nps prepared by nanoprecipitation with a blend ratio of PEG_5000_-PLGA/PLGA 70/30, possessing the smaller particle size, was selected to assess the antimicrobial activity [59].

Antimicrobial assays were performed against methicillin-resistant *S. aureus* since FS10 is structurally related to the RIP for which it is well-known the ability to inhibit *S. aureus* biofilm pathogenesis interfering in the QS. Moreover, this bacterium is responsible for hospital and community-acquired infections difficult to treat because of the growing multiresistance to antimicrobials and because of its capability to forming biofilm. Methicillin-resistant *S. aureus* (MRSA) belongs to the so-called ESKAPE cluster which consists of the most dangerous microorganisms isolated in hospitals [60].

Even if FS10, FS10-loaded-Nps and empty Nps showed no significant antibacterial activity versus *S. aureus* ATCC 43300 at the tested concentrations (Table 3), interestingly, MICs and MBCs of FS10-loaded-Nps were found lower than those of the free FS10. These results revealed that the therapeutic efficiency of FS10 improved upon nanoparticulate formation. The enhanced antibacterial activity of FS10-loaded Nps could be mainly related to the nanosized dimensions responsible for improved cellular uptake and thus to more significant damage in the cell membrane of the bacteria, or to the protection of encapsulated FS10 which may act more efficiently escaping from clearance, inactivation, or degradation processes.

### 3.8. Antibiofilm Activity of FS10, FS10-Loaded-Nps, and Empty Nps versus S. aureus ATCC 43300

The MBIC was performed to evaluate the possible effect of FS10, FS10-loaded-Nps, and empty Nps. Results revealed that the tested samples inhibit *S. aureus* ATCC 43300 biofilm formations at a concentration higher than 256 µg/mL as reported in Table 4.

**Figure 9:**
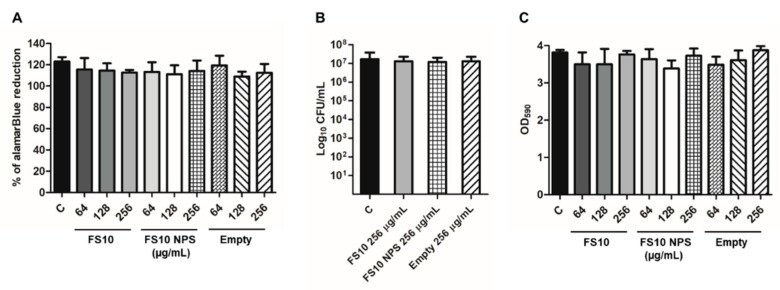
Determination of the MBIC of FS10, FS10-loaded-Nps, and empty Nps through the alamarBlue assay (**A**), the CFU count (**B**), and the Crystal Violet assay (**C**). C, control biofilm. The results represent the mean of two independent experiments performed in triplicate. C (ANOVA + Dunnet’s Multiple comparison test).

The evaluation of the biofilm formation was performed by using the three assays mentioned in the methods section. The alamarBlue assay showed the lack of activity of the three compounds in inhibiting biofilm formation. Nevertheless, the CFU count was carried out to determine even a possible minimum variation in the value of *S. aureus* ATCC 43300 biofilm vitality. In detail, the CFU count was carried out by starting by the wells corresponding to the biofilms treated with the major concentrations of the three compounds corresponding to 256 µg/mL. As reported in Figure 9B the CFU count of the biofilms formed at 256 µg/mL of each compound didn’t show any difference when compared to untread control biofilm. The Crystal Violet assay was performed to evaluate *S. aureus* biofilm biomass formed with FS10, FS10-Loaded-Nps, and empty Nps. As reported in Figure 9C the biofilm biomass, expressed as OD590 absorbance had the same value both in the treated and the untreated control biofilms.

### 3.9. Hemolytic Activity

FS10 Hemolytic activity was previously assessed [29]. Furthermore, empty Nps and FS10-loaded Nps were evaluated for their ability to disrupt mice erythrocyte membranes in comparison with melittin. Even at a higher concentration tested, a cellular disruption inferior to 8% was observed with all the tested samples.

## 4. Conclusions

Polymeric nanoparticles loaded with a QS-inhibiting peptide for the eradication of the *S. aureus* biofilm were prepared by double emulsion and nanoprecipitation technique. The different FS10-loaded nanoparticles, composed of different weight ratios of PEG_5000_-PLGA and PLGA 25 kDa uncapped, were investigated for their physico-chemical properties, drug release kinetics, and antimicrobial efficacy. Taking together the obtained results a good peptide loading was achieved increasing the pH to 9.7. Different Nps showed a similar peptide release up to 21 h. Furthermore, the antimicrobial activity of FS10-loaded-Nps versus *S. aureus* ATCC 43300 was observed at lower concentrations compared to the free FS10 while the antibiofilm activity was found at a concentration higher than 256 µg/mL. These results prove that such formulations may be employed as a drug delivery platform for hydrophilic therapeutics to protect them from a fast degradation, thus improving their accumulation in the target site. Further experiments will be necessary to evaluate the capability of the FS10-loaded-Nps alone and in combination with other antimicrobials, to inhibit the biofilm developed by ESKAPE species grown alone and in combination with each other.

## Figures and Tables

**Table 1:** Characteristics of the synthesized PEG-PLGA block copolymer.

Polymer	Feed Molar L/G Ratio (%)	Polymer L/G Ratio ^a^	M_w_ (kDa) ^b^	M_n_ (kDa)	Theoretical M_n_ (kDa)	PDI ^b^	PEG (wt%)	*T_g_* (°C)
MePEG_5000_-PLGA	50:50	49:51	17.6	17.5 ^a^ 9.6 ^b^	18.0	1.8	28.5 ^a^	1

^a^ NMR, and ^b^ GPC analysis.

**Table 2:** Stability of empty and FS10-loaded PEG-PLGA Nps at 4 °C up to 14 days.

Time (Days)	Size (nm)		PDI	
	Empty	FS10-Loaded Nps	Empty	FS10-Loaded Nps
0	97.1 ± 1.3	105.1 ± 1.2	0.144	0.205
1	99.45 ± 1.5	112.5 ± 2.3	0.232	0.242
2	131.1 ± 0.5	141.3 ± 1.1	0.142	0.271
4	169.4 ± 2.5	130.4 ± 0.8	0.202	0.253
6	158.9 ± 1.3	168.1 ± 0.3	0.258	0.278
8	169.5 ± 3.5	181.4 ± 0.2	0.300	0.307
10	164.7 ± 2.1	186.5 ± 2.1	0.307	0.329
14	189.4 ± 0.3	171.4 ± 2.6	0.332	0.378

**Table 3:** The determination of the MIC and MBC of FS10, FS10-loaded-Nps, and empty Nps versus *S. aureus* ATCC 43300. The results represent the mean of two independent experiments performed in triplicate.

	MIC (µg/mL)	MBC (µg/mL)
FS10	>256	>256
FS10-loaded-Nps	>128	>128
Empty Nps	>512	>512

**Table 4:** Determination of the MBIC of FS10, FS10-loaded-Nps, and empty Nps versus *S. aureus* ATCC 43300. Results represent the mean of two independent experiments performed in triplicate.

	MBIC (µg/mL)
FS10	>256
FS10-loaded-Nps	>256
Empty Nps	>256

## Data Availability

Data are contained in the article and Supplementary Materials.

## Supplementary Materials

The following supporting information can be downloaded at: https://www.mdpi.com/article/10.3390/pharmaceutics14091821/s1, Figure S1: 1H-NMR spectrum of PEG5000-PLGA in deuterated chloroform. δ= 1.5 (m, 3H, -CH3), 4.6 (m, 4H, O-CH2-CH2), 4.8–4.9 (m, 2H, O-CH2-C(O)O), 5.1–5.2 (m, 1H, -CH-CH3); Figure S2: GPC chromatogram of the synthesized diblock copolymer; Figure S3: Computational pKa calculation (ChemAxon pKa Predictor); Figure S4: Computational pKa calculation (ChemAxon pKa Predictor); Figure S5: FS10 stability studies performed in human plasma at 37 °C.
