# Supplementary material for: Preparation, Characterization, and Biological Evaluation of a Hydrophilic Peptide Loaded on PEG-PLGA Nanoparticles
Source: Pharmaceutics. 2022 Aug 29;14(9):1821. doi: 10.3390/pharmaceutics14091821 (PMC9506305; doi:10.3390/pharmaceutics14091821)
Supplement: Supplementary file 1 [file pharmaceutics-14-01821-s001.zip › pharmaceutics-1802639-supplementary.pdf]

Supplementary Materials

Preparation, Characterization, and Biological Evaluation of a Hydrophilic Peptide Loaded on PEG-PLGA Nanoparticles

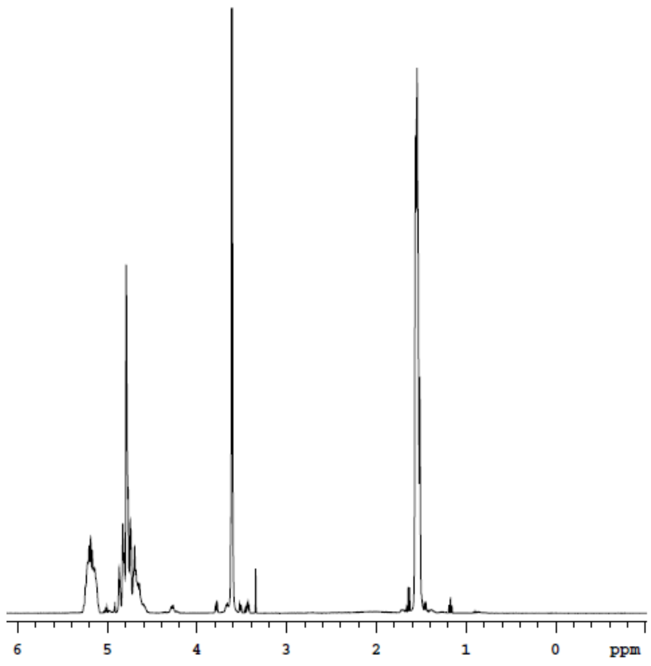

Figure S1. ^{1}H-NMR spectrum of PEG_{5000}-PLGA in deuterated chloroform. δ= 1.5 (m, 3H, -<u>CH_3</u>), 4.6 (m, 4H, O-<u>CH_2-CH_2</u>), 4.8-4.9 (m, 2H, O-<u>CH_2</u>-C(O)O), 5.1-5.2 (m, 1H, <u>-CH</u>-CH_3).

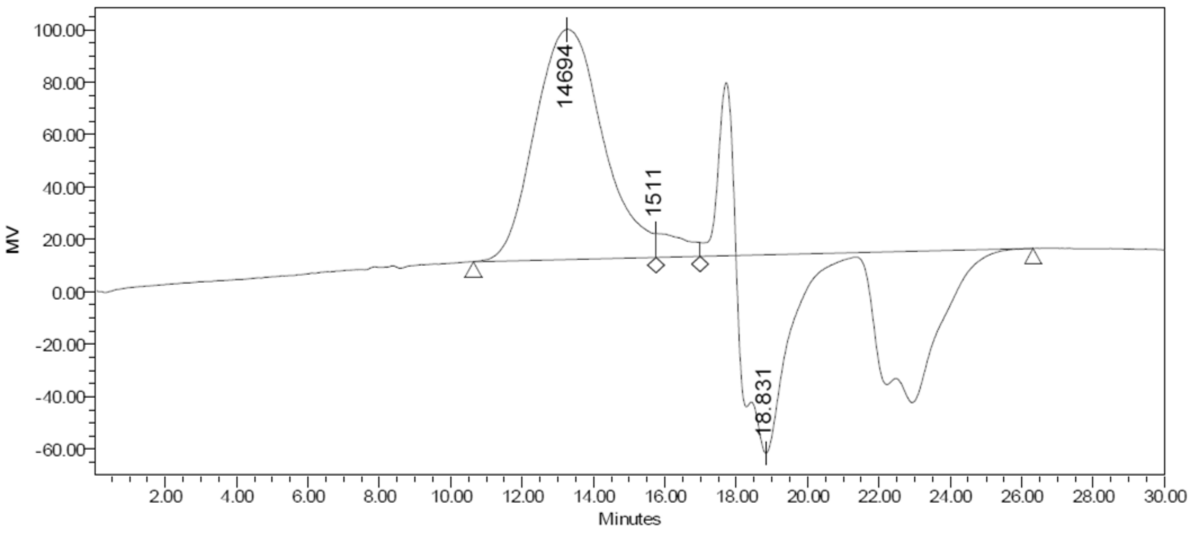

Figure S2. GPC chromatogram of the synthetized diblock copolymer.

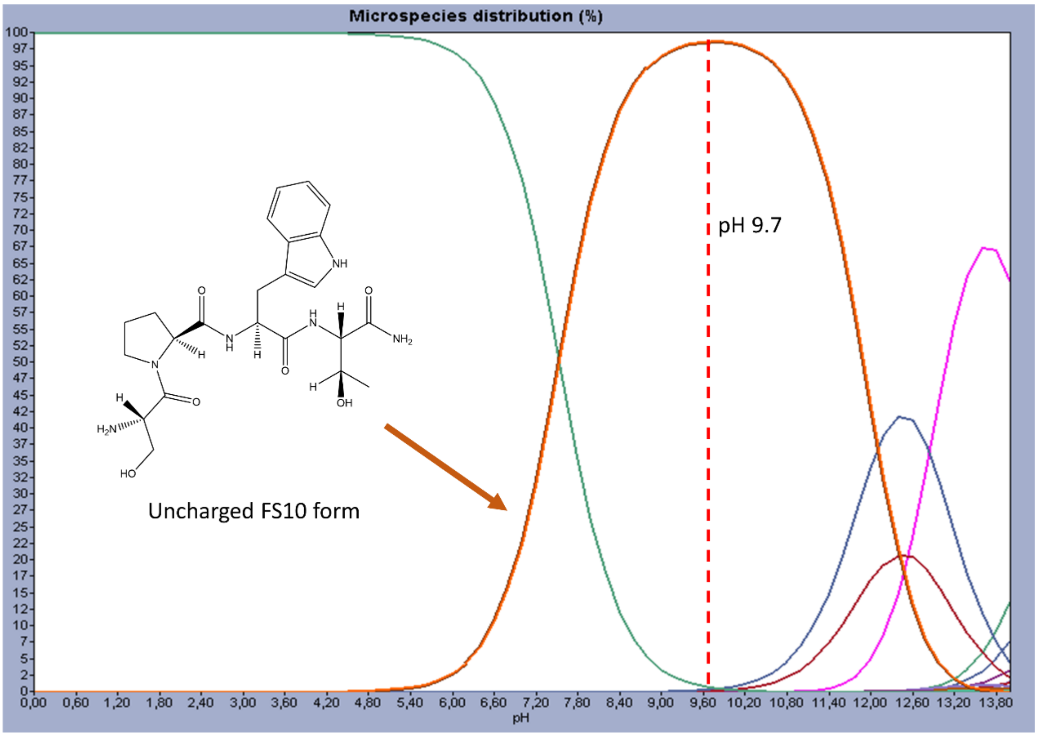

Figure S3. Computational pKa calculation (ChemAxon pKa Predictor).

Computational calculations

Computational calculations were performed by the software ChemAxon pKa Predictor - Version 15.4.13.0. In particular, this software calculated all possible FS10 structures, charged and uncharged, and assigned the percentage of the macrospecies distribution related to the pH of the solution. The percentage of the unique uncharged form of FS10 is 98.7% at pH 9.7 (Figure S4).

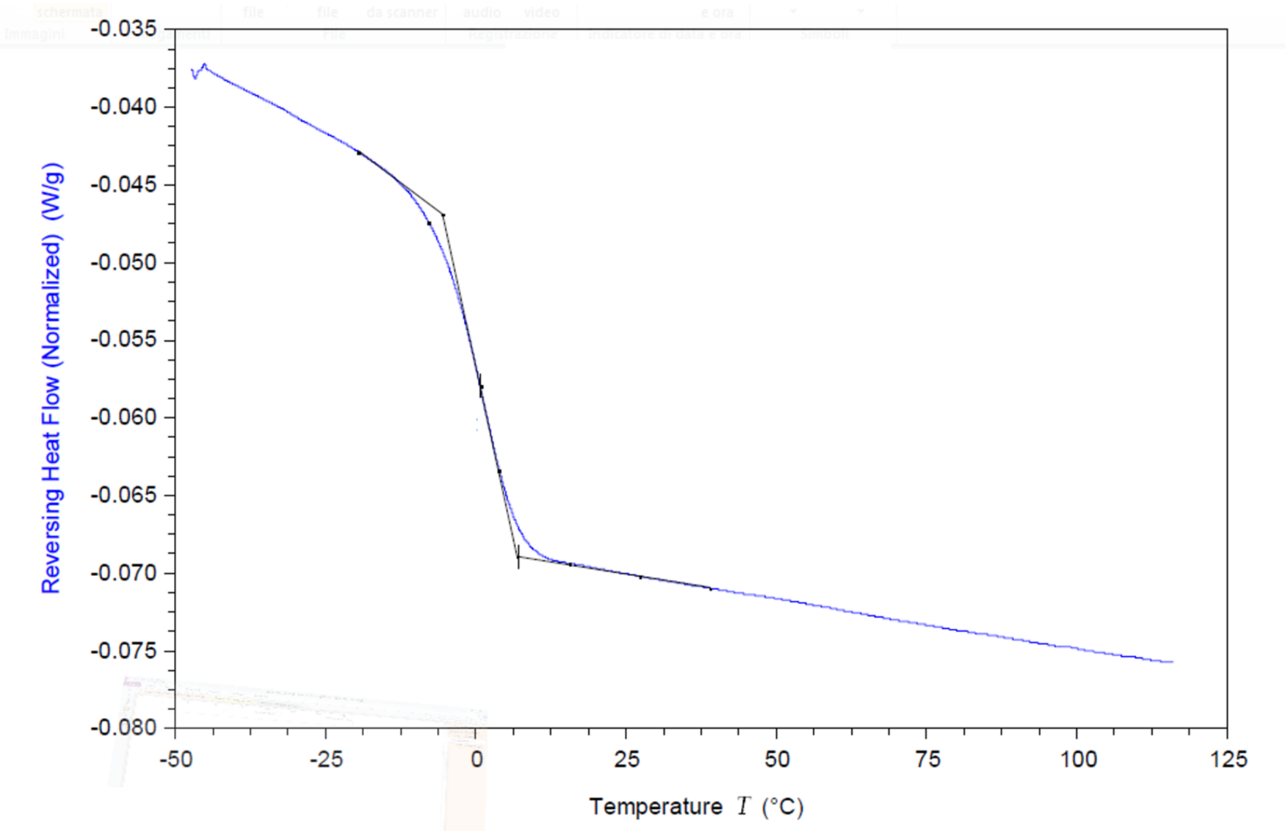

Figure S4. Computational pKa calculation (ChemAxon pKa Predictor).

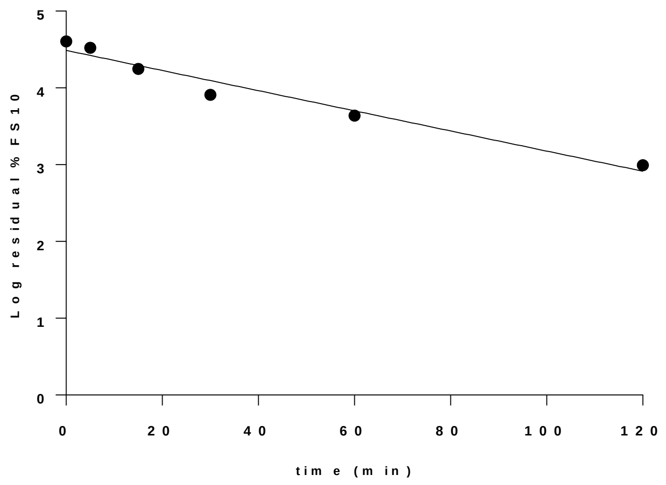

Figure S5. FS10 stability studies performed in human plasma at 37 °C.

Experimental stability studies

In vitro stability studies were performed in human plasma (3H Biomedical, Uppsala, Sweden). FS10 (1 mg) was dissolved in 1 mL of a mixture composed by plasma/PBS buffer in a ratio 80:20 v/v, maintained at 37 °C, under stirring. At fixed time points, aliquots of 100 μL were withdrawn and mixed with 100 μL of ACN, in order to precipitate plasma proteins and stop the enzymatic reaction. After centrifuge (5 min, 12000 rpm and 4 °C), the supernatant was analysed by HPLC.
